# Differences in neutralizing antibody sensitivities and envelope characteristics indicate distinct antigenic properties of Nigerian HIV-1 subtype G and CRF02_AG

**DOI:** 10.1186/s12985-024-02394-y

**Published:** 2024-06-29

**Authors:** Lindsay Wieczorek, David Chang, Eric Sanders-Buell, Michelle Zemil, Elizabeth Martinez, Jesse Schoen, Agnes-Laurence Chenine, Sebastian Molnar, Brittani Barrows, Kultida Poltavee, Man E. Charurat, Alash’le Abimiku, William Blattner, Michael Iroezindu, Afoke Kokogho, Nelson L. Michael, Trevor A. Crowell, Julie A. Ake, Sodsai Tovanabutra, Victoria R. Polonis, on behalf of the AFRICOS and TRUST/RV368 Study Groups, Danielle Bartolanzo, Alexus Reynolds, Katherine Song, Mark Milazzo, Leilani Francisco, Shauna Mankiewicz, Steven Schech, Alexandra Golway, Badryah Omar, Tsedal Mebrahtu, Elizabeth Lee, Kimberly Bohince, Ajay Parikh, Jaclyn Hern, Emma Duff, Kara Lombardi, Michelle Imbach, Leigh Anne Eller, Hannah Kibuuka, Michael Semwogerere, Prossy Naluyima, Godfrey Zziwa, Allan Tindikahwa, Hilda Mutebe, Cate Kafeero, Enos Baghendaghe, William Lwebuge, Freddie Ssentogo, Hellen Birungi, Josephine Tegamanyi, Paul Wangiri, Christine Nabanoba, Phiona Namulondo, Richard Tumusiime, Ezra Musingye, Christina Nanteza, Joseph Wandege, Michael Waiswa, Evelyn Najjuma, Olive Maggaga, Isaac Kato Kenoly, Barbara Mukanza, Jonah Maswai, Rither Langat, Aaron Ngeno, Lucy Korir, Raphael Langat, Francis Opiyo, Alex Kasembeli, Christopher Ochieng, Japhet Towett, Jane Kimetto, Brighton Omondi, Mary Leelgo, Michael Obonyo, Linner Rotich, Enock Tonui, Ella Chelangat, Joan Kapkiai, Salome Wangare, Zeddy Bett Kesi, Janet Ngeno, Edwin Langat, Kennedy Labosso, Joshua Rotich, Leonard Cheruiyot, Enock Changwony, Mike Bii, Ezekiel Chumba, Susan Ontango, Danson Gitonga, Samuel Kiprotich, Bornes Ngtech, Grace Engoke, Irene Metet, Alice Airo, Ignatius Kiptoo, John Owuoth, Valentine Sing’oei, Winne Rehema, Solomon Otieno, Celine Ogari, Elkanah Modi, Oscar Adimo, Charles Okwaro, Christine Lando, Margaret Onyango, Iddah Aoko, Kennedy Obambo, Joseph Meyo, George Suja, Yakubu Adamu, Nnamdi Azuakola, Mfreke Asuquo, Abdulwasiu Bolaji Tiamiyu, Samirah Sani Mohammed, Ifeanyi Okoye, Sunday Odeyemi, Aminu Suleiman, Lawrence Umejo, Onome Enas, Miriam Mbachu, Ijeoma Chigbu-Ukaegbu, Wilson Adai, Felicia Anayochukwu Odo, Rabi Abdu, Rosemary Akiga, Helen Nwandu, CHisara Okolo, Ndubuisis Okeke, Zahra Parker, Asogwa Ugochukwu Linus, Concilia Amaka Agbaim, Tunde Adegbite, Nkenchiere Harrison, Adewale Adelakun, Ekeocha Chioma, Victoria Idi, Rachel Eluwa, Jumoke Nwalozie, Igiri Faith, Blessing Okanigbuan, Achugwo Emmanuel, Nkiru Nnadi, Ndubuisi Rosemary, Uzoegwu Amaka Natalie, Obende Theresa Owanza, Falaju Idowu Francis, Jacintal Elemere, Obilor Ifeoma Lauretta, Edward Akinwale, Inalegwu Ochai, Lucas Maganga, Emmanuel Bahemana, Samoel Khamadi, John Njegite, Connie Lueer, Abisai Kisinda, Jaquiline Mwamwaja, Faraja Mbwayu, Gloria David, Mtasi Mwaipopo, Reginald Gervas, Doroth Mkondoo, Nancy Somi, Paschal Kiliba, Gwamaka Mwaisanga, Johnisius Msigwa, Hawa Mfumbulwa, Peter Edwin, Willyhelmina Olomi, Manhattan Charurat, Aka Abayomi, Sylvia Adebajo, Stefan Baral, Charlotte Gaydos, Fengming Hu, Jennifer Malia, Rebecca Nowak, Uchenna Ononaku, Sheila Peel, Habib Ramadhani, Merlin Robb, Cristina Rodriguez-Hart, Elizabeth Shoyemi, Abdulwasiu Tiamiyu, Sandhya Vasan

**Affiliations:** 1 U.S. Military HIV Research Program, CIDR, Walter Reed Army Institute of Resarch, Silver Spring, MD USA; 2 Henry M. Jackson Foundation for Advancement of Military Medicine, Bethesda, MD USA; 3 SEARCH, Insititute of HIV Research and Innovation (IHRI), Bangkok, Thailand; 4 Institute of Human Virology, University of Maryland School of Medicine, Baltimore, MD USA; 5 HJF Medical Research International, Abuja, Nigeria; 6 Center for Infectious Disease Research, Walter Reed Army Institute of Resarch, Silver Spring, MD USA; 7 Present address: Office of AIDS Research, National Institutes of Health, Rockville, MD 20852 USA; 8 Present address: Integrated Biotherapeutics, Rockville, MD 20850 USA; 9 Present address: Lentigen Technology, Gaithersburg, MD 20878 USA

**Keywords:** HIV, Envelope (Env), Antibody, Nigeria, West Africa, Subtype G, CRF02_AG, Neutralization

## Abstract

The magnitude of the HIV-1 epidemic in Nigeria is second only to the subtype C epidemic in South Africa, yet the subtypes prevalent in Nigeria require further characterization. A panel of 50 subtype G and 18 CRF02_AG Nigerian HIV-1 pseudoviruses (PSV) was developed and envelope coreceptor usage, neutralization sensitivity and cross-clade reactivity were characterized. These PSV were neutralized by some antibodies targeting major neutralizing determinants, but potentially important differences were observed in specific sensitivities (eg. to sCD4, MPER and V2/V3 monoclonal antibodies), as well as in properties such as variable loop lengths, number of potential N-linked glycans and charge, demonstrating distinct antigenic characteristics of CRF02_AG and subtype G. There was preferential neutralization of the matched CRF/subtype when PSV from subtype G or CRF02_AG were tested using pooled plasma. These novel Nigerian PSV will be useful to study HIV-1 CRF- or subtype-specific humoral immune responses for subtype G and CRF02_AG.

**Supplementary Information:**

The online version contains supplementary material available at 10.1186/s12985-024-02394-y.

## Introduction

Nigeria has the second-largest global HIV epidemic, with approximately 2 million people living with HIV (PLWH) in 2022 [1]. HIV prevalence is lower than in other sub-Saharan African countries, determined by the 2018 Nigeria HIV/AIDS Indicator and Impact Survey (NAIIS) to be 1.4% overall [2]. However, concentrated sub-epidemics exist among key populations such as men who have sex with men and, due to the country’s large population, Nigeria accounts for over 40% of all new HIV infections in Western and Central Africa [3, 4]. While incidence in West Central Africa has decreased since 2010, there are still many barriers to controlling the epidemic. A 2017 survey of PLWH in this region found only 48% were aware of their status, 40% were receiving antiretroviral therapy (ART) and only 29% were virally suppressed [3]. Rates of vertical transmission outpace other regions at 20.2% [2, 3]. Childhood testing and treatment also require further advancement; in Nigeria, only 26% of children living with HIV receive ART [3]. Additionally, pretreatment HIV drug resistance prevalence of up to 20.5% was recently reported, highlighting the need for additional treatment and prevention modalities [5, 6].

Effective vaccines may be most beneficial in a setting of limited treatment availability and adherence. Vaccine efficacy of 31.2% was observed in the RV144 clinical trial in Thailand, where subtypes B and CRF01_AE are prevalent [7]. Correlates analysis of this study identified that envelope (Env)-elicited antibodies, specifically against the V1V2 loop, correlated inversely with infection risk [8–10]. However, the design of a broadly effective vaccine is complicated in part by sequence diversity in the Env protein. Env diversity of up to 20% within the same subtype and 35% between different subtypes has been reported [11]. To be effective, vaccine-induced antibodies must cross-react with the Envs of circulating viral strains. The HIV genetic diversity in West Africa is extensive and increasing [12, 13]. HIV-1 subtype G, CRF02_AG and their recombinants were shown to be the major circulating variants in Nigeria, with 50% of all HIV-1 strains in Nigeria containing a subtype G gp120 Env [14]. Further understanding of subtype G and CRF02_AG is needed to better develop methods to control infections in this region.

While subtype G and CRF02_AG are prominent in West Africa, they are expanding in other regions such as Europe, particularly in France. Subtype G and CRF02_AG Envs from both continents have not been extensively characterized [15–21]. As of November 2023, 485 subtype G *env* sequences exist in the Los Alamos National Laboratory (LANL) database for 154 unique individuals worldwide, and 635 CRF02_AG *env* sequences exist for 314 unique individuals worldwide. Only 64 of these subtype G and 26 of the CRF02_AG infections were from Nigeria. For comparison, 59,990 subtype B *env* sequences exist for 4,795 unique individuals worldwide. In addition to the comparative paucity of genetic information, little is known about the humoral immune responses elicited during subtype G and CRF02_AG infections, and the comparative neutralization sensitivities of G and CRF02_AG Envs have not been well defined. Understanding vulnerable neutralizing determinants of the G and CRF02_AG Envs, as well as cross-clade reactivities, will be important for understanding the immunobiology of subtype G and CRF02_AG.

In this study, we developed and characterized a novel panel of subtype G and CRF02_AG Envs isolated from 10 Nigerian people with HIV-1 subtype G and 5 with CRF02_AG infection. Envs were generated from serum or plasma from participants in three Nigerian cohorts, including the African Cohort Study (AFRICOS), the Recruiting Acute Cases of HIV (REACH) study, and the TRUST/RV368 cohort. Envs expressed as pseudoviruses (PSV), were evaluated for coreceptor usage, neutralization sensitivity to a panel of neutralizing monoclonal antibodies (NmAbs), and cross-clade reactivities using polyclonal reagents.

## Materials and methods

### Cohort descriptions

Nigerian subtype G and CRF02_AG plasma samples were obtained from three Nigerian cohort studies, including the African Cohort Study (AFRICOS), the Recruiting Acute Cases of HIV (REACH) study and TRUST/RV368 (Supplementary Table 1). AFRICOS is an ongoing longitudinal study conducted by the U.S Military HIV Research Program (MHRP) in Abuja and Lagos, Nigeria beginning in 2013 as previously described [22]. REACH was a longitudinal study conducted by the Institute of Human Virology in Abuja and Jos, Nigeria between 2003 and 2010, as previously described [23]. TRUST/RV368 is an ongoing prospective observational cohort study conducted by the Institute of Human Virology and MHRP in Abuja and Lagos, Nigeria beginning in 2013 [24, 25]. All samples were collected and provided under the respective IRB-approved protocols. Samples were selected for use in this study based on availability and estimated infection >1 year.

### Identification of Subtype G and CRF02_AG samples

Extracted RNA from AFRICOS participant plasma were used in the Multi-Region Hybridization Assay (MHA) to identify subtype G and/or CRF02_AG probe reactivity across seven gene regions of the HIV-1 genome as previously described [14]. Samples that possessed a high proportion of subtype G reactivity (and/or G/CRF02_AG dual reactivity) were subjected to full genome sequencing at near-endpoint dilution, and subtyping was performed by phylogenetic analysis (NCBI Genotyping Tool, and alignment with African subtype G sequences retrieved from the Los Alamos National Laboratory HIV Database). Samples from five participants in the AFRICOS cohort (MUW100708A, MUW101091A, MUW101273A, MUW101769A, and MUW104349A) were identified as pure subtype G infections. Samples from these five participants, in conjunction with another five subtype G samples from participants in the REACH cohort (SC13, SC20, SC21, SC26, and SC62) for which subtype and sequence data (of other clones) have been previously published [23], were used for envelope clone generation. CRF02_AG samples came from 5 CRF02_AG infected individuals identified by full genome sequencing, 4 from the REACH cohort (SC12, SC28, SC29 and SC30) and 126652 was identified from the TRUST/RV368 Cohort.

### HIV+ plasma pools

Plasma pools were generated using equal volumes of 5–10 individual plasmas for each subtype, as previously described [26]. Pooled plasmas were from chronically infected individuals with viruses that were determined to be pure subtype by full-length genome or full-length Env sequencing. Subtype A plasmas were from Kenya and Tanzania, subtype B plasmas from the United States, subtype C plasmas from Tanzania, subtype D plasmas were from Kenya and Uganda, subtype G and CRF02_AG plasmas were from Nigeria, and CRF01_AE plasmas were from Thailand.

### Generation of envelope gene clones for pseudovirus production

Envelope glycoprotein (gp160) genes were amplified by single genome amplification and cloned into eukaryotic expression vectors for use in pseudovirus assays, as previously described [26, 27]. Briefly, first round half-length single genome amplification PCR products were produced from cDNA generated from plasma or serum viral RNA. From the (3’-) half-length DNA template, the *env* PCR product was produced using the primers BH4minus (5’-TAGGCATTTCCTATGGCAGGAAGAAG; HXB2 5958-5983 nt) and BH2NOENZ2ACE (5’-GTCTCGAGATACTGCTCCTACTC; HXB2 8904-8882 nt), and Platinum Taq DNA polymerase (Invitrogen/Thermo). The amplicon was gel purified using the QIAquick Gel Extraction Kit (Qiagen), cloned into pcDNA3.1/V5-His-TOPO eukaryotic cloning vector (Invitrogen/Thermo), and transformed into either STBL2 or STBL4 cells (Invitrogen/Thermo). Plasmid DNA were purified from bacterial cultures using the Qiagen Plasmid Maxi Kit (Qiagen). Env sequence was verified by comparison with original SGA-derived amplicon. The median number of amino acid differences between the cloned envelope genes and the uncloned envelope amplicon was 0 (mean 0.75, range 0-3). Env function was confirmed in the TZM-bl pseudovirus assay.

### Pseudovirus preparation and titration

Preparation of pseudoviruses (PSV) was performed by transfecting 5 × 10^6^ HEK293T cells with 8 μg of *env* expression plasmid and 24 μg of an *env*-deficient HIV-1 backbone vector (pSG3ΔEnv), using X-tremeGENE 9 transfection reagent (Roche). Culture supernatants were harvested at day 4 and stored at − 80 °C. PSV stock viral input infectivity was evaluated with the assistance of Biomek NXP liquid handler (Beckman Coulter, Indianapolis, Indiana). PSV stocks were serial diluted 3-fold for a total of ten dilutions in 96-well plates and then 12.5µL quadruplicates were transferred to 384-well culture plates. Culture medium was added to each well to a final volume of 25µL. Each well then received 3 × 10^3^ TZM-bl cells suspended in 25µL of growth medium containing 40µg/mL DEAE-dextran. After a 48-hour incubation at 37 °C in a humidified 5% CO_2_–95% air environment, culture medium was removed from each well to final volume of 30µL. Reconstituted Bright-Glo Luciferase Assay Substrate (Promega Corp, Madison, WI) was added to all wells at a 1:2 dilution(30µL). Relative light units were detected with the SpectraMax Paradigm Microplate Reader (Molecular Devices, Sunnyvale, California, USA). Wells producing relative luminescence units (RLU) > 2.5x background were scored as positive.

### Coreceptor determination by the GHOST cell assay

The GHOST cell infection assay was used to determine co-receptor usage of viral stocks. Parental, CXCR4-expressing, or CCR5-expressing GHOST cells (NIH AIDS Reagent Program, Germantown, MD) were cultured in 24-well plates at 1x10^5^ cells per well. Cells were infected with undiluted PSV in the presence of 20 µg/ml of Polybrene infection reagent (MilliporeSigma, Billerica, MA) for 4 h. Cells were washed, cultured for 2 days and then harvested and fixed with 2% paraformaldehyde. The percentage of infected cells expressing green fluorescent protein was measured by flow cytometry analysis using an LSRII cytometer and FACSDIVA software (Becton-Dickinson, San Jose, CA). Post-acquisition analysis was conducted with FlowJo software (FlowJo, LLC, Ashland, OR). PSVs were designated as using the CXCR4 or CCR5 receptor if the ratio to control negative (RTCN) was >10; RTCN= (MFI x %pos)_infected_ / (MFI x %pos)_uninfected,_ as previously described [28]. Assay controls included an uninfected negative control, murine leukemia virus as a positive control, and CCR5 and CXCR4 utilizing PSVs as cell line controls.

### High-throughput pseudovirus (PSV) neutralization assay

NmAb IC_50_s or titers were determined using TZM-bl cells in a high-throughput assay utilizing robotic liquid handling. The following PSVs were assessed: a multi-subtype tier 1 PSV panel, a CRF01_AE tier 2 transmitted-founder PSV panel and murine leukemia virus (MuLV) (nonspecific control). Serum was diluted 1:5 in growth medium and serially diluted using the Biomek NXP liquid handler (Beckman Coulter, Indianapolis, Indiana, USA). Titered serum (12.5ml/well) was transferred to 384-well culture plates and incubated with an equal volume of PSV for 45 min at 37°C. TZM-bl cells (3x10^3^ cell/well) with DEAE-dextran (40µg/ml) were added to each well and incubated for an additional 48 h. Relative light units were detected with the SpectraMax Paradigm Microplate Reader (Molecular Devices, Sunnyvale, California, USA) using the Bright-Glo Luciferase Assay System (Promega Corporation, Madison, Wisconsin, USA). Neutralization dose–response curves were fitted by nonlinear regression using the LabKey Server, and the final titer is reported as the reciprocal of the dilution of serum necessary to achieve 50% neutralization (50% inhibitory dose).

#### Data availability

Sequence data that support the findings of this study are currently being deposited into GenBank. Data are shown within the manuscript or supplemental information files, upon publication, neutralization data will be deposited into the LANL CATNAP database. Data are available from the corresponding author upon reasonable request.

## Results

### Development of an HIV-1 subtype G and CRF02_AG Env panel

Subtype G and CRF02_AG *envs* were generated by Single Genome Amplification (SGA) using serum or plasma collected from three Nigerian cohorts, including the AFRICOS, REACH, and TRUST/RV368 cohorts (Supplemental Table S1). While acute and early HIV-1 infection samples were collected in rare instances, the majority of the *envs* were cloned from Fiebig stage VI or chronic infection. This was a result of sample availability and also allowed for better comparison with our previously established multi-subtype chronic Env panel that represented 10 Envs each from subtypes A, B, C, D, and CRF01_AE and CRF02_AG [26]. Ninety-two subtype G *env* sequences were produced and forty-nine CRF02_AG *env* sequences were generated. HIV-1 subtype was determined using the full genome sequences. The *envs* were expressed as PSV by co-transfection of 293T cells with the *env* plasmid and a plasmid expressing the HIV backbone (pSG3ΔEnv); infectivity was evaluated using the TZMbl cell line. To represent the infectious quasispecies present in each participant, Envs that were sufficiently infectious (RLUs for virus only ≥10x RLUs for cell only) were evaluated for neutralization sensitivity in this study, including 50 Subtype G Envs (10 participants, 3-7 clones per participant) and 18 CRF02_AG Envs (5 participants, 2-6 clones per participant) (Supplemental Table S1).

**Fig. 1:**
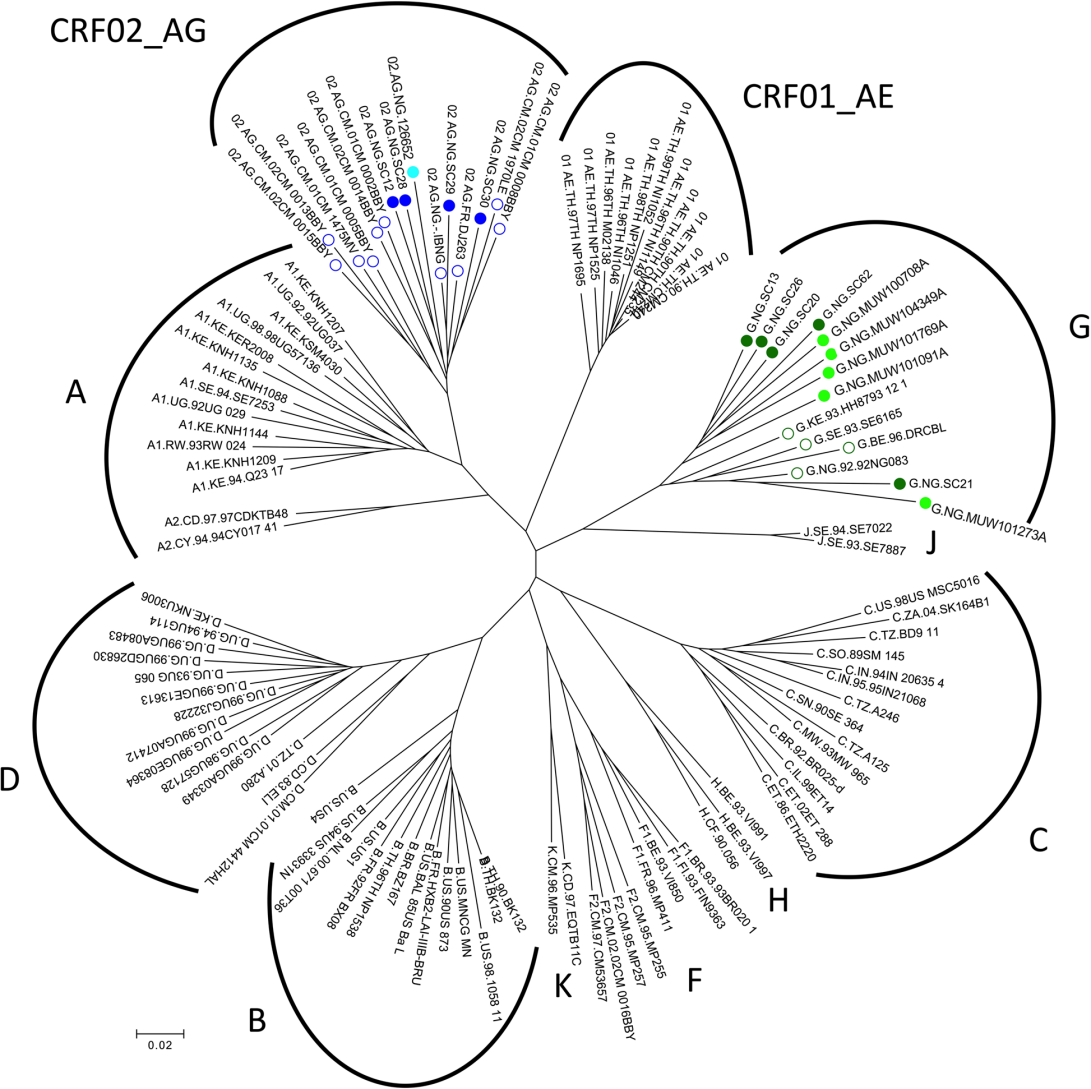
Phylogenetic analysis of Nigerian subtype G and CRF02_AG envs*.* Relationship of HIV-1 subtype G and CRF02_AG *env* nucleotide sequences with reference subtype *envs*. Subtype G *envs* used in this study are represented as dark green (REACH) or light green (AFRICOS) circles. CRF02_AG Envs are dark blue (REACH) or light blue (TRUST/RV368); one *env* variant is represented for each individual. Subtype G and CRF02_AG reference strains are shown as open circles

Phylogenetic analysis of the *env* genes was used to examine the genetic relatedness between the Nigerian subtype G and CRF02_AG panel with *envs* from our multi-subtype panel and other subtype reference strains (Fig. 1). Nigerian CRF02_AG *env*s are most closely related to subtype A *envs*; the gp120 and external gp41 region are derived from subtype A in CRF02_AG [29] and subtype G *env* strains cluster distinctly from CRF02_AG.

**Fig. 2:**
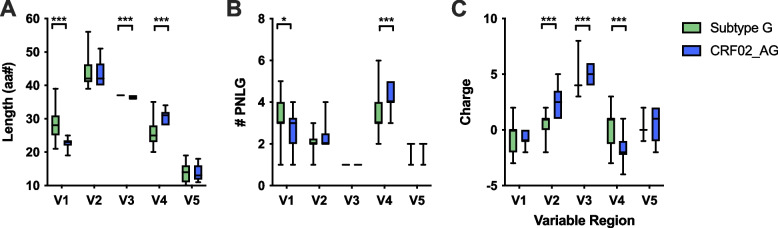
Antigenic characteristics of Nigerian subtype G and CRF02_AG Envs. **A** Variable loop length, **B**) number of potential N-linked glycosylation sites (PNLG), and **C**) overall charge are shown for subtype G (green) and CRF02_AG (blue) Envs. Statistical differences were determined using Mann Whitney U Test; * = *p*<0.05, ** = *p*<0.005, *** = *p*<0.0005

To evaluate evidence for potential differences in antigenic properties of subtype G and CRF02_AG, the length, number of potential N-linked glycosylation sites (PNLG), and charge were evaluated for variable loops V1, V2, V3, V4 and V5 (Fig. 2). Subtype G Envs had longer V1 regions with more V1 PNLG sites than CRF02_AG Envs (*p*<0.0001 and *p*=0.0083, respectively, Fig. 2A). CRF02_AG Envs had longer V4 loops with more V4 PNLG sites than subtype G Envs (both *p*<0.0001, Fig. 2B). CRF02_AG Envs had more positively charged V2 and V3 regions, and more negatively charged V4 regions, than did subtype G Envs (Fig 2C), indicating potential antigenic differences between Nigerian subtype G and CRF02_AG. Accurate representation of the number of participants for subtype G and CRF02_AG can be seen in Supplemental Fig. S1. For each participant, the mean values for all clones were plotted for each antigenic property. Since the numbers compared reduced from 50 vs 18 (clones) down to 10 vs 5 (participants), the statistical significance was lost in some cases, but the trends in Fig. S1 remain the same as shown in Fig. 2.

### Analysis of subtype G and CRF02_AG Env coreceptor usage

Coreceptor usage of the 50 subtype G and 18 CRF02_AG PSVs was evaluated using the GHOST cell assay (Supplemental Table S1). The GHOST cell lines express CD4 and either CCR5 or CXCR4. Envs were classified as CCR5- or CXCR4-utilizing if a ratio to control negative (RTCN) score for the respective cell line was ≥10. For one CRF02_AG Env and the three subtype G Envs that were poorly infectious in GHOST cells (RTCN <10 for both cell lines), tropism was predicted using the V3 Env sequences and the geno2pheno[coreceptor] algorithm [30]; Envs were classified as utilizing CCR5 if the false-positive rate (FPR) was >10% (Supplemental Table S1, bold and italic font). Amongst the 18 CRF02_AG PSV, there were no CXCR4 Envs, and 6/50 (12%) subtype G Envs were dual tropic (Table S1).

### Neutralization sensitivity of subtype G and CRF02_AG HIV-1

**Fig. 3:**
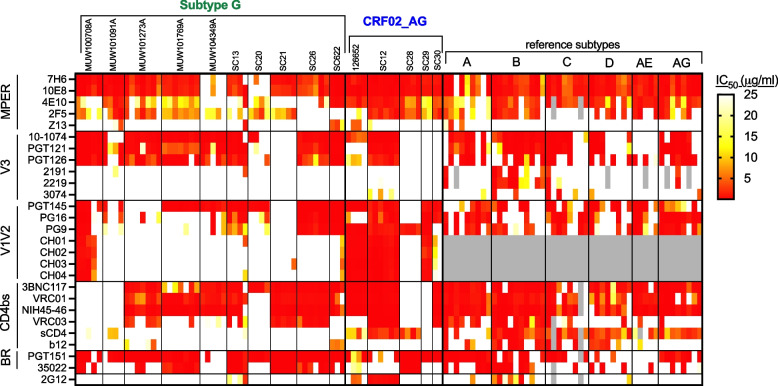
Heat map of the subtype G and CRF02_AG PSV neutralization IC_50_s compared with reference panel PSV. The IC_50_s for each NmAb tested against individual participant PSV were used to generate a heat map. The vertical black lines separate the clones for each participant and participant IDs, as well as clone subtypes or CRFs are indicated at the top of the figure. As indicated by the scale, stronger red coloring indicates more potent neutralization, and grey shading denotes not tested

**Fig. 4:**
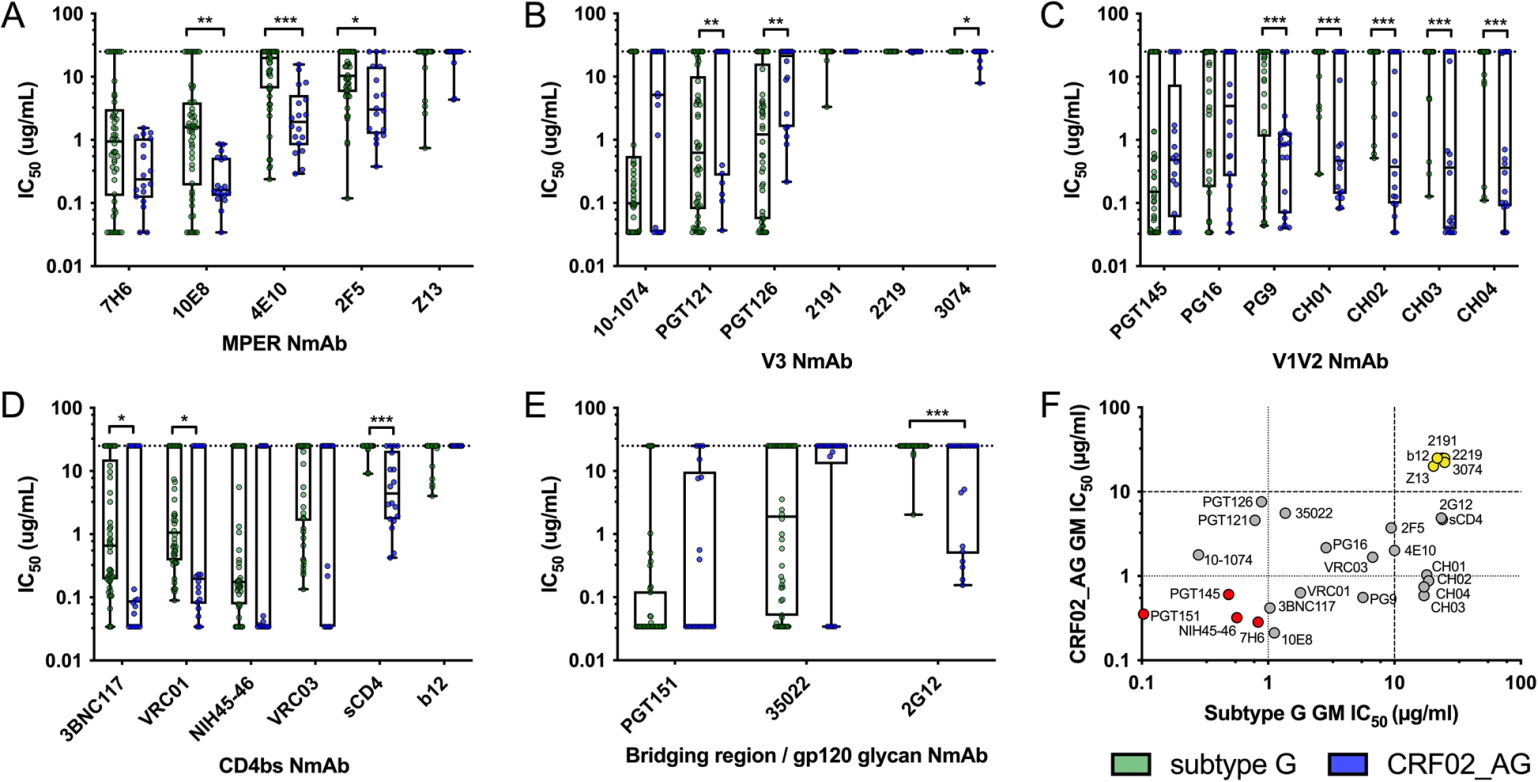
Neutralization profiles for subtype G versus CRF02_AG using individual NmAbs. NmAbs targeting the **A**) MPER, **B**) V3, **C**) V1V2, **D**) CD4bs and **E**) bridging regions and gp120 glycan were tested against individual PSV and the IC_50_ values graphed. Statistical differences between Subtype G and CRF02_AG PSV were determined using Mann Whitney U Test; * = *p*<0.05, ** = p<0.005, *** = *p*<0.0005, as indicated. **F** The NmAb GM IC_50_ was determined for all subtype G and CRF02_AG PSV and plotted to reflect relative NmAb potencies and differences between HIV subtype G and CRF02_AG. The dotted and dashed lines indicate NmAb neutralization potency of GM IC_50_ = 1 μg/ml or 10 μg/ml, respectively. NmAbs shown in red are potently neutralizing against both subtype G and CRF02_AG; NmAbs shown in yellow were weakly neutralizing against both

To evaluate potential differences in neutralization sensitivities of these Nigerian subtype G and CRF02_AG PSV, they were evaluated in the TZMbl neutralization assay using reagents that included soluble CD4 (sCD4) and 27 NmAbs targeting oligo-mannose (2G12) and the major neutralizing determinants of HIV Env, specifically, the membrane proximal external region (MPER), V3, V1V2, CD4 binding site (CD4bs), and the bridging region (BR) (Figs. 3 and 4, Supplemental Figs. S2 and S3). For direct comparison of other HIV-1 subtypes with this panel of 68 sucbtype G and CRF02_AG PSV, the neutralization sensitivities of PSV from additional subtypes and CRF01_AE were evaluated. Reference PSV (9 A, 10 B, 9 C, 9 D, 5 CRF01_AE, and 8 older CRF02_AG from other countries) were utilized from a previously assembled chronic multi-subtype international panel [26]. The heat map of 3,194 individual IC_50_ values is shown in Fig. 3. There were 210 NmAb/virus pairs for which IC_50_ values were not performed (shown in grey shading). Four V2/V3-conformational antibodies (CH01-CH04) were evaluated only against the new panel of 50 subtype G and 18 CRF02_AG PSV (Fig. 3, grey shading, not tested). CRF02_AG Envs were significantly more sensitive to these 4 V2/V3-specific NmAbs, as shown in Fig. 3, Fig 4C and most prominently in the supplemental Fig. S2. The IC_50_s for each NmAb against all clones from each participant were averaged to generate a geometric mean IC_50_ (GM IC_50_) and those data are represented in the heat map in Fig. S2. The clone averaged data show similar patterns as observed in Fig. 3, but the differences are more notable when the averaged data per participant are mapped. The poor CH01-CH04 neutralization of the subtype G clones for 9/10 participants, in comparison to potent neutralization (by CH01, CH02, CH03 and CH04) of all clones from 3/5 CRF02_AG infected participants can readily be visualized in Fig. S2. Further, using the PG9 conformational V1V2 NmAb, for 4/5 CRF02 participants, the GM IC_50_s were potent (<5 ug/ml), while only 4/10 subtype G participant clone GM IC_50_s showed some potency for PG9 (Fig. S2). In contrast, clones from 9/10 subtype G participants showed strong neutralization by V3 NmAbs, but clones from only 2/5 CRF02_AG infected participants showed V3 NmAb neutralization (Fig. S2). These data indicate distinct differences in the V2 versus V3 accessibility of CRF02_AG Envs, as compared with subtype G Envs.

To investigate the statistical significance of the differences between subtype G and CRF02_AG PSV neutralization observed in the heat maps, sensitivities were directly compared and IC_50_s graphed for each individual NmAb (Fig. 4). Significantly higher sensitivity of CRF02_AG Envs to MPER-specific NmAbs can be seen for 3 (4E10, 10E8 and 2F5) of the 5 MPER mAbs tested (Fig. 4A). While all CRF02_AG Envs were sensitive to 4E10 and 10E8 NmAbs, 4E10 resistance was observed for 22/50 (44%) subtype G Envs (Fisher’s exact test *p*=0.0003) and 10E8 resistance was observed for 8/50 subtype G Envs (16%) (Fig. 3 and Fig. 4A), indicating potential differences in MPER accessibility. For all CD4bs reagents, the CRF02_AG PSV were more sensitive than G PSVs, with significant differences observed for 3BNC117, VRC01 and sCD4; sCD4 resistance was observed for 46 subtype G Envs (92%), and only 4 (22%) of the CRF02_AG Envs (Fisher’s *p*=0.0001) (Fig. 4D). CRF02_AG Envs were significantly more sensitive to the gp120 mannose-dependent NmAb, 2G12, and resistance was observed for 47/50 subtype G Envs (94%), versus 10 (56%) of the CRF02_AG Envs (*p*=0.0006). Interestingly, the only instances where subtype G Envs were more sensitive than CRF02_AG Envs was for the glycan-dependent V3 NmAbs, PGT121 and PGT126. As shown in the heat maps, subtype G was much more sensitive to the V3 NmAbs overall, as compared to CRF02_AG. The clone averaged data per participant (expressed as GM IC_50_s) are graphed in Fig. S3; the trends were similar to those observed in Fig. 4.

To contrast the averaged IC_50_s for these NmAbs against subtype G versus CRF02_AG and highlight potential overall sensitivity differences, geometric mean IC_50_s were calculated for each NmAb and graphed in comparison to determine within which Env target the Subtype G and CRF02_AG PSVs were most consistently sensitive or resistant (Fig. 4F). Subtype G and CRF02_AG Envs were both universally sensitive (GMT <1 µg/ml) to neutralization by PGT151 (bridging region), PGT145 (V1V2), NIH45-46 (CD4bs) and MPER 7H6 (shown in red circles in Fig. 4F). Subtype G and CRF02_AG Envs were both universally resistant (GMT >20 µg/ml) to neutralization by Z13 (MPER), b12 (CD4bs), and 2219, 3074 and 2191 (V3) (shown in yellow circles, Fig. 4F). No specific Env domain exhibited enhanced susceptibility for both, highlighting a lack of similarity in antigenicity. Overall, the subtype G Envs showed more resistance to NmAb neutralization; for 13/27 NmAbs tested, subtype G IC_50_s were significantly higher (Fig. 4), indicating greater resistance).

### Cross-subtype reactivity of subtype G and CRF02_AG PSVs and plasma

**Fig. 5:**
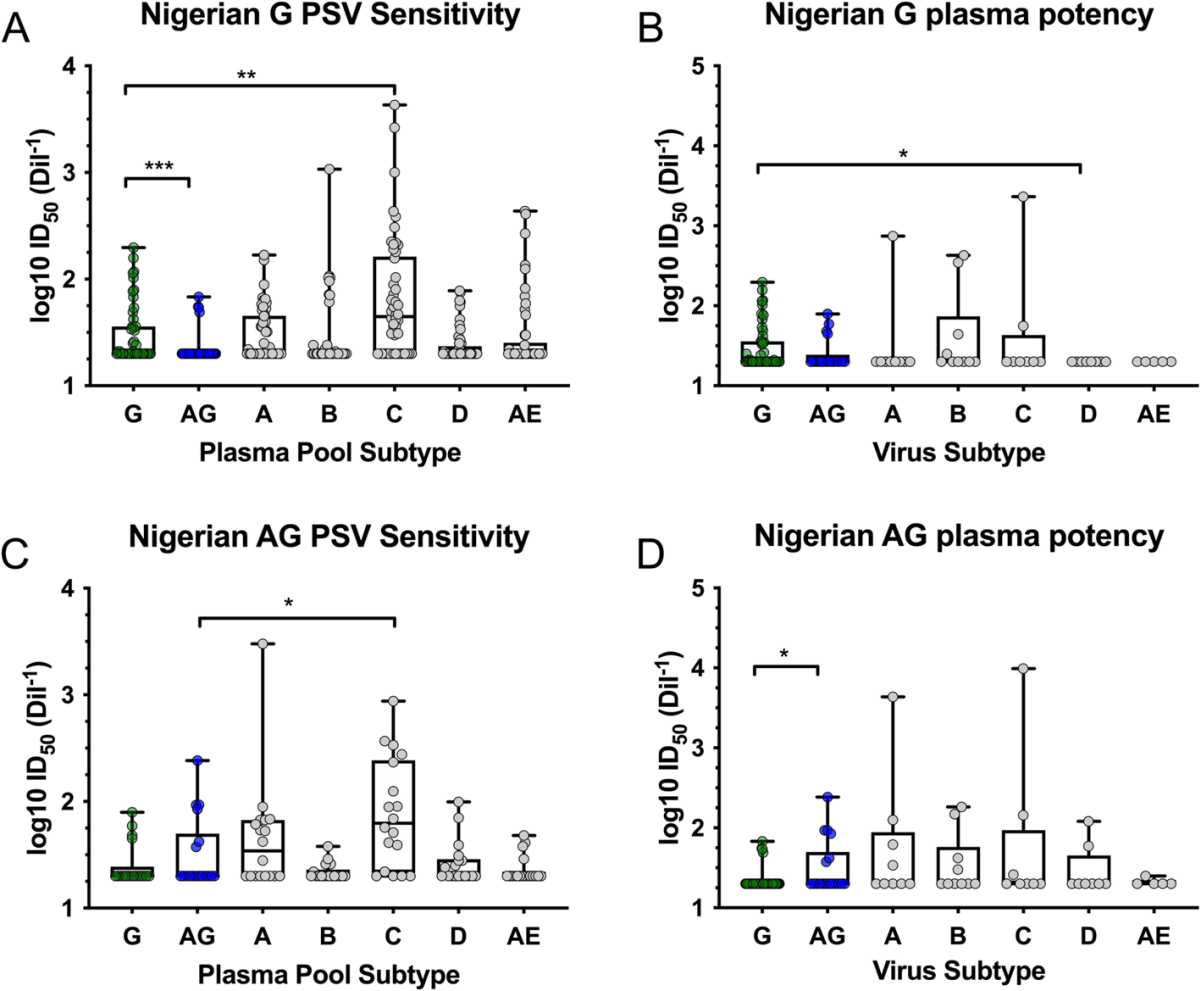
Cross-clade reactivity of subtype G and CRF02_AG PSV and HIV+ plasma. Plasma pool neutralization was evaluated in the TZMbl neutralization assay against the subtype G and CRF02_AG PSV and reference strains for subtype A, B, C, D and CRF02_AG. Sensitivity to neutralization by plasma pools from pure HIV-1 subtypes/CRFs was determined for all **A**) subtype G and **C**) CRF02_AG PSV. Plasma potency was determined against PSVs from different HIV subtypes/CRFs for the **B**) subtype G and **D**) CRF02_AG plasma pools. Statistical differences were determined using Mann Whitney U Test; * = *p*<0.05, ** = *p*<0.005, *** = *p*<0.0005

Using previously characterized plasma pools, the cross-reactivity of Nigerian subtype G and CRF02_AG strains was evaluated to better understand polyclonal neutralization sensitivities. Pools were used to determine overall clade-specific neutralization and to avoid the variabilities that can be observed when using individual plasmas. Neutralization profiles of all PSV were evaluated using plasma pools from subtypes A, B, C, D, and G, as well as CRF01_AE and CRF02_AG (Fig. 5A and 5C). Nigerian subtype G and CRF02_AG plasma pools were generated by pooling equal volumes of plasma from 5 participants with chronic HIV-infection from the REACH and AFRICOS cohorts. In the same manner, plasma pools representing other subtypes were generated previously, each including plasma from 5 to 10 pure-subtype chronically infected individuals [26].

The potency of neutralization by the subtype C plasma pool was statistically greater than that of the respective matched plasma pool for both subtype G PSVs (*p*=0.0007, Fig. 5A) and CRF02_AG PSVs (*p*=0.009; Fig. 5C). These results are consistent with previous data reporting the neutralization breadth of subtype C-specific IgG from Tanzania [26]. The subtype G plasma pool was more potent against subtype G PSVs as compared to the subtype CRF02_AG plasma pool (*p*<0.0001, Fig. 5A), indicating potentially stronger clade-specific neutralization. Additionally, when the potency of the G and CRF02_AG plasma pools was evaluated against viruses from the 5 subtypes and 2 CRFs, the CRF02_AG plasma pool was more potent against CRF02_AG PSV as compared to G PSV (*p*=0.0058, Fig. 5D). The G plasma pool also trended towards better neutralization of the G viruses (Fig. 5B).

The subtype G plasma pool showed the best coverage of subtype G Envs, with minimal to no breadth observed against PSVs from other subtypes (Fig. 5B). The CRF02_AG plasma pool showed the greatest coverage against CRF02_AG and subtypes A, B and C, with minimal cross-neutralization observed against PSVs from subtypes G and CRF01_AE (Fig. 5D). Additionally, correlations were observed between plasma breadth and potency for subtype G (*p*=0.1659, *r*=0.6055) and CRF02_AG (*p*=0.0341, *r*=0.8214). Strong correlations were also observed between the magnitude and frequency of within subtype PSVs neutralized for subtype G (*p*=0.0202, *r*=0.8727) and CRF02_AG (*p*=0.0095, *r*=0.9009) (data not shown), as expected. Data obtained when the ID_50_ values for PSV from each participant were averaged and graphed by participant showed the same trends as data shown in Fig. 5 (data not shown).

## Discussion

While HIV acquisition rates in West Central Africa have declined, additional prevention measures may be required to effectively control the epidemic, as subtype G and CRF02_AG are also prominent and expanding in areas outside of Africa. Subtype G and B/G recombinant HIV strains circulate not only in Nigeria, but also in Western Europe, particularly in Spain and Portugal [17, 18, 31]. Reports have identified subtype G infections in China and Russia, indicating a greater need for surveillance of this subtype [15, 32]. Introduction of these subtypes to Europe and Asia has led to expansion of local epidemics and to an increase in population viral diversity [33, 34]. The current prevalence and global expansion of HIV subtype G and CRF02_AG highlights the need to better represent these subtypes in future vaccine designs and in studies characterizing antigenic and immunogenic properties of HIV-1 subtypes. That approximately 50% of the gp120 Envs in Nigeria are subtype G [14], combined with our observation that for nearly 50% of the NmAbs tested, subtype G is significantly less sensitive to neutralization than CRF02_AG, warrants improved understanding of the biology and immunology of these subtypes.

CRF02_AG is also becoming more widespread, expanding in Spain, France, Brazil and Russia [19, 20, 35–37]. Recently, a panel of 33 CRF02_AG PSVs from France was characterized using 11 NmAbs [21] and distinct differences were observed between our findings using West African CRF02_AG and the French CRF02_AG Envs. While they observed relative resistance to PGT145 and PG9 amongst the French PSV, the Nigerian PSV in our panel showed sensitivity to both PGT145 (14/18 sensitive) and PG9 (16/18 sensitive). Similar to our panel, the French CRF02_AG PSV demonstrated strong susceptibility to CD4bs NmAbs 3BNC117, NIH45-46, and VRC01, as well as to the MPER NmAb 10E8 [21]. Both CRF02_AG panels showed resistance to V3-directed NmAbs (particularly 10-1074). Unfortunately, they did not test the four V2/V3 conformational CH01-CH04 NmAbs [38], where strong sensitivity was observed for the Nigerian CRF02_AG PSV (Figs. 3 and 4).

The difference observed in V1V2 NmAb reactivity in these two populations could be indicative of regional differences evolving in the strains circulating in Europe versus West Africa and this warrants further study. However, the French panel incorporated mainly early infection PSV (estimated to be from 24-115 days post-infection), whereas our panels were derived mostly from Fiebig stage VI or chronic infection. It is interesting to note that in an extensive analysis of 219 PSV for impact of age, clade and geography on neutralization profiles, Hraber et al. found no evidence that transmitted/founder viruses are more susceptible to neutralization and are therefore easier targets for vaccination than chronic viruses [39]. In that study, 112/219 viruses were from intermediate or late HIV infection stages. Chronic infection viruses, such as those presented herein, were also used to define the antigenic distinction between subtype B and CRF01_AE, found to be co-circulating in Thailand [40]. This observation remained the same when Envs from earlier in infection were incorporated [39]. We postulate that data obtained using our current chronic panel suggest a similar antigenic distinction between subtype G and CRF02_AG co-circulating in Nigeria. It will be very important to study the acute and early infection samples that are now possible to collect through our AFRICOS protocol [41].

Many of the current HIV-1 vaccine approaches focus on eliciting neutralizing antibodies targeting important epitopes of the HIV-1 Env [42, 43]. Variations of the Env protein are currently being evaluated as vaccine immunogens, including monomeric or trimeric Env proteins, Env-domain scaffold proteins, and Env peptides. Env epitopes targeted by NmAbs are represented in these antigens with the expectation that antibodies with similar specificities and function will be elicited. Major Env domains targeted by NmAbs include the V1V2, V3, CD4bs, MPER and the bridging region. These vulnerable Env targets were evaluated in this study; subtypes G and CRF02_AG Envs were relatively sensitive to NmAbs targeting the Env bridging region and/or V1V2. Bridging region NmAbs (PGT151 and 35O22) bind both the gp120 and gp41 Env subunits, with preferential binding to native-like Env trimers [44]. V1V2 NmAbs also recognize quaternary epitopes at the V2 domain of the trimer apex [45–47] and conformational domains that can include V3 [38]. This indicates the probable importance of trimeric or conformationally relevant antigens for elicitation of protective antibody responses in this region. The considerable resistance of the subtype G PSV to inhibition by sCD4 and b12 indicates a difference in how these Envs may engage CD4. Also, differences in V1V2 and V3 NmAb sensitivities strongly suggest differential exposure of these variable loops in CRF02_AG versus subtype G. The data shown are limited to the NmAbs analyzed, and many of the available NmAbs are derived from subtype B infections (eg. b12, 2G12). However, the number of non-B specific NmAbs is now growing due to acute infection studies [48, 49] and other longitudinal studies [50, 51]; some of these newer NmAbs were included here.

Beyond applications in vaccine approaches, several mAbs are now being evaluated as therapeutics in clinical trials, including NmAbs targeting the MPER (10E8v4), V3 (10-1074), V1V2 (PGDM1400) and CD4bs (3BNC117, VRC01, N6) [52]. Of these available NmAbs, 10E8 may represent the greatest coverage for both subtypes G (84% PSVs sensitive, 1.12 µg/ml GMT) and CRF02_AG (100% PSVs sensitive, 0.21 µg/ml GMT), as suggested in this study. Additionally, tri-specific NmAb cocktails have been developed, simultaneously targeting the MPER, V1V2 and CD4bs; these cocktails have shown improvements over use of single NmAbs in conferring complete immunity in non-human primate challenge studies [53]. The tri-specific NmAb therapeutic approach may improve coverage against distinct HIV-1 subtypes. Another observation for NmAbs that has been made by several groups, including ours [54] is the increasing resistance of more contemporary viruses to several NmAbs over the decades of the epidemic. In our present study, we used the available samples from chronic infection within the available cohorts in Nigeria. We considered the span of years included for the Envs from each subtype/CRF and felt that, although not optimal, the span or mixture of temporal sampling was about the same for each subtype/CRF. Thus, for subtype G, clones were included from years 2007-2014, and for CRF02_AG, clones were included for 2006-2015, rather similar time frames. In addition, within subtype G we assessed all 27 mAbs for IC_50_s against the clones (*N*=22) from the participants who gave samples in 2007-08 and compared them with the IC_50_s against the clones (*N*=28) from the participants who gave samples in 2013-14. The *p*-values by Mann-Whitney U were not significant, except for 1/27 mAbs (3BNC117, moderately significant) (data not shown). We conclude that, although temporal differences in sampling may exert some influence and this is a caveat of the study, we found no significant evidence of change in neutralization sensitivity over the time span used in this study.

Fc-mediated effector mechanisms may also be important, or required, to inhibit or control HIV infection in vivo [55]. Additional experiments will be required to characterize the sensitivity of subtype G and CRF02_AG viral strains to ADCC and effector-specific functional activities mediated by antibodies with these important non-neutralizing functions. Studies using samples from acute infection will also be critical.

Development of an effective HIV vaccine may need to be focused on specific HIV subtypes circulating in the target region due to the high genetic diversity, or on conserved Env epitopes to elicit immune responses that will be protective against diverse circulating strains. To better understand the cross-reactivity of co-circulating subtype G and CRF02_AG Env strains, we evaluated plasma pool neutralization against the new PSV panels. We observed poor cross-reactivity between the Nigerian subtype G and CRF02_AG (Fig. 5). Similarly, poor cross-reactivity between major co-circulating subtypes has also been observed in Thailand, where subtypes B and CRF01_AE, and their recombinants, are prevalent [26, 56]. These results need to be taken into consideration when designing vaccine Env antigens, as one antigen subtype alone may not be effective at providing broad protection. Both co-circulating subtypes were included in the vaccination strategy for the moderately efficacious RV144 Thai trial [7, 57]. Inclusion of antigens representing the major and distinct circulating HIV subtypes may increase the breadth required for protective immunity. It may be necessary to include both subtype G and CRF02_AG in vaccines to achieve strain coverage and protection for this West African sub-region.

Results from this study will contribute to increasing the information needed to better understand the immunobiology of the strains co-circulating in Nigeria. The novel panel of 68 chronic PSVs and *env* sequences described here will significantly add to the available reagents for use in research involving these understudied viruses. Surveillance of the phylogenetic and neutralization differences in contemporary circulating subtype G and CRF02_AG strains should be utilized to better design vaccine immunogens and to select appropriate therapeutic NmAbs. Development of an HIV vaccine effective against subtype G and CRF02_AG would significantly advance the goals to reduce HIV incidence in Nigeria and end the AIDS epidemic as a public health threat.

### Supplementary Information

Supplementary Material 1: Table S1.Panel of 50 subtype G Envs and 18 CRF02_AG Envs from Nigeria*. *The participants and subtype/CRF for each env clone in the panel are listed at the left of the table. The years in which the samples were collected, the samples source, the amino acid sequence for the tip of the V3 loop and the coreceptor usage (R5 denotes CCR5 usage and X4/R5 denotes dual tropic or use of both CXCR4 and CCR5) are indicated. Bold and italic font indicate PSV for which the Geno2Pheno tool was used to predict coreceptor usage.

Supplementary Material 2: Fig. S1. Antigenic characteristics of Nigerian subtype G and CRF02_AG Envs shown by averaged values per participant. A geometric mean value for all sequences for each participant was derived and graphed for: A) Variable loop length, B) number of potential N-linked glycosylation sites (PNLG), and C) overall charge, and presented for subtype G (green) and CRF02_AG (blue) Envs. Statistical differences were determined using Mann Whitney U Test;    * = *p*<0.05, ** = *p*<0.005, *** = *p*<0.0005.

Supplementary Material 3: Fig. S2. Heat map of the NmAb neutralization of subtype G and CRF02_AG GM IC_50_s compared with reference panel PSV IC_50_s.  The IC_50_s for each NmAb against all of the clones from each participant were used to generate a GM IC_50_ and a heat map of these values was generated. The clone subtypes or CRFs are indicated at the top of the figure and the NmAbs and Env domains are listed to the left. As indicated by the scale, stronger red coloring indicates more potent neutralization; grey shading denotes not tested.

Supplementary Material 4: Fig. S3. Neutralization profiles for subtype G versus CRF02_AG using individual NmAbs and GM IC50s for all clones per participant. NmAbs targeting the A) MPER, B) V3, C) V1V2, D) CD4bs and E) bridging regions and gp120 glycan were tested and GM IC50s were generated for all clones for each participant and graphed. Statistical differences between Subtype G and CRF02_AG PSV were determined using Mann Whitney U Test; *= *p*<0.05, ** = *p*<0.005, *** = *p*<0.0005, as indicated. F) The NmAb GM IC_50_ was determined for all subtype G or CRF02_AG PSV and plotted to reflect relative NmAb potencies and differences between HIV subtype G and CRF02_AG. The dotted and dashed lines indicate NmAb neutralization potency of GM IC_50_ = 1 mg/ml or 10 mg/ml, respectively. NmAbs shown in red circles are potently neutralizing against both subtype G and CRF02_AG; NmAbs shown in yellow were weakly neutralizing against both.

## Data Availability

Sequence data that support the findings of this study are currently being deposited into GenBank. Data are shown within the manuscript or supplementary information files, upon publication, neutralization data will be deposited into the LANL CATNAP database. Data are available from the corresponding author upon reasonable request.

## References

[1] Onovo AA, Adeyemi A, Onime D, Kalnoky M, Kagniniwa B, Dessie M (2023). Estimation of HIV prevalence and burden in Nigeria: a Bayesian predictive modelling study. EClinicalMedicine.

[2] National Summary Sheet - Prelimiary Findings. National HIV/AIDS Indicator and Impact Survey, 2019.

[3] UNAIDS. UNAIDS Data 2022. 2022.

[4] Keshinro B, Crowell TA, Nowak RG, Adebajo S, Peel S, Gaydos CA (2016). High prevalence of HIV, chlamydia and gonorrhoea among men who have sex with men and transgender women attending trusted community centres in Abuja and Lagos, Nigeria. J Int AIDS Soc.

[5] Crowell TA, Danboise B, Parikh A, Esber A, Dear N, Coakley P (2021). Pretreatment and Acquired Antiretroviral Drug Resistance Among Persons Living With HIV in Four African Countries. Clin Infect Dis.

[6] Crowell TA, Kijak GH, Sanders-Buell E, O'Sullivan AM, Kokogho A, Parker ZF (2019). Transmitted, pre-treatment and acquired antiretroviral drug resistance among men who have sex with men and transgender women living with HIV in Nigeria. Antivir Ther..

[7] Rerks-Ngarm S, Pitisuttithum P, Nitayaphan S, Kaewkungwal J, Chiu J, Paris R (2009). Vaccination with ALVAC and AIDSVAX to prevent HIV-1 infection in Thailand. N Engl J Med.

[8] Haynes BF, Gilbert PB, McElrath MJ, Zolla-Pazner S, Tomaras GD, Alam SM (2012). Immune-correlates analysis of an HIV-1 vaccine efficacy trial. N Engl J Med.

[9] Rerks-Ngarm S, Pitisuttithum P, Excler JL, Nitayaphan S, Kaewkungwal J, Premsri N (2017). Randomized, Double-Blind Evaluation of Late Boost Strategies for HIV-Uninfected Vaccine Recipients in the RV144 HIV Vaccine Efficacy Trial. J Infect Dis.

[10] Zolla-Pazner S, deCamp AC, Cardozo T, Karasavvas N, Gottardo R, Williams C (2013). Analysis of V2 antibody responses induced in vaccinees in the ALVAC/AIDSVAX HIV-1 vaccine efficacy trial. PLoS One.

[11] Gaschen B, Taylor J, Yusim K, Foley B, Gao F, Lang D (2002). Diversity considerations in HIV-1 vaccine selection. Science (New York, NY).

[12] Lihana RW, Ssemwanga D, Abimiku A, Ndembi N (2012). Update on HIV-1 diversity in Africa: a decade in review. AIDS Rev.

[13] Vidal N, Koyalta D, Richard V, Lechiche C, Ndinaromtan T, Djimasngar A (2003). High genetic diversity of HIV-1 strains in Chad, West Central Africa. J Acquir Immune Defic Syndr.

[14] Heipertz RA, Ayemoba O, Sanders-Buell E, Poltavee K, Pham P, Kijak GH (2016). Significant contribution of subtype G to HIV-1 genetic complexity in Nigeria identified by a newly developed subtyping assay specific for subtype G and CRF02_AG. Medicine (Baltimore).

[15] Li T, Lan Y, Li F, Li H, Deng H, Liu Y, et al. Characterization of Four Nearly Full-Length Genomic Sequences of HIV-1 Subtype G Identified in Guangdong Province, China. AIDS Res Hum Retroviruses. 2019. 10.1089/aid.2019.0025. Epub 2019/02/23. PubMed PMID: 30793935. 10.1089/AID.2019.0025 30793935

[16] Ngo-Giang-Huong N, Huynh THK, Dagnra AY, Toni TD, Maiga AI, Kania D (2019). Prevalence of pretreatment HIV drug resistance in West African and Southeast Asian countries. J Antimicrob Chemother.

[17] Thomson MM, Delgado E, Manjon N, Ocampo A, Villahermosa ML, Marino A (2001). HIV-1 genetic diversity in Galicia Spain: BG intersubtype recombinant viruses circulating among injecting drug users. Aids.

[18] Esteves A, Parreira R, Venenno T, Franco M, Piedade J, Germano De Sousa J (2002). Molecular epidemiology of HIV type 1 infection in Portugal: high prevalence of non-B subtypes. AIDS Res Hum Retroviruses.

[19] Chaix ML, Seng R, Frange P, Tran L, Avettand-Fenoel V, Ghosn J (2013). Increasing HIV-1 non-B subtype primary infections in patients in France and effect of HIV subtypes on virological and immunological responses to combined antiretroviral therapy. Clin Infect Dis.

[20] Brand D, Moreau A, Cazein F, Lot F, Pillonel J, Brunet S (2014). Characteristics of patients recently infected with HIV-1 non-B subtypes in France: a nested study within the mandatory notification system for new HIV diagnoses. J Clin Microbiol..

[21] Stefic K, Bouvin-Pley M, Essat A, Visdeloup C, Moreau A, Goujard C, et al. Sensitivity to Broadly Neutralizing Antibodies of Recently Transmitted HIV-1 Clade CRF02_AG Viruses with a Focus on Evolution over Time. J Virol. 2019;93(2). 10.1128/jvi.01492-18. Epub 2018/11/09. PubMed PMID: 30404804; PubMed Central PMCID: PMCPMC6321924. 10.1128/JVI.01492-18 PMC6321924 30404804

[22] Ake JA, Polyak CS, Crowell TA, Kiweewa F, Semwogerere M, Maganga L, et al. Noninfectious Comorbidity in the African Cohort Study (AFRICOS). Clin Infect Dis. 2018. 10.1093/cid/ciy981. Epub 2018/11/27. PubMed PMID: 30476001; PubMed Central PMCID: PMCPMC6669288. 10.1093/cid/ciy981 PMC6669288 30476001

[23] Charurat M, Nasidi A, Delaney K, Saidu A, Croxton T, Mondal P (2012). Characterization of acute HIV-1 infection in high-risk Nigerian populations. J Infect Dis.

[24] Charurat ME, Emmanuel B, Akolo C, Keshinro B, Nowak RG, Kennedy S (2015). Uptake of treatment as prevention for HIV and continuum of care among HIV-positive men who have sex with men in Nigeria. J Acquir Immune Defic Syndr.

[25] Baral SD, Ketende S, Schwartz S, Orazulike I, Ugoh K, Peel SA (2015). Evaluating respondent-driven sampling as an implementation tool for universal coverage of antiretroviral studies among men who have sex with men living with HIV. J Acquir Immune Defic Syndr.

[26] Brown BK, Wieczorek L, Sanders-Buell E, Rosa Borges A, Robb ML, Birx DL (2008). Cross-clade neutralization patterns among HIV-1 strains from the six major clades of the pandemic evaluated and compared in two different models. Virology.

[27] Salazar-Gonzalez JF, Salazar MG, Keele BF, Learn GH, Giorgi EE, Li H (2009). Genetic identity, biological phenotype, and evolutionary pathways of transmitted/founder viruses in acute and early HIV-1 infection. J Exp Med.

[28] Vodros D, Tscherning-Casper C, Navea L, Schols D, De Clercq E, Fenyo EM (2001). Quantitative evaluation of HIV-1 coreceptor use in the GHOST3 cell assay. Virology..

[29] Carr JK, Salminen MO, Albert J, Sanders-Buell E, Gotte D, Birx DL (1998). Full genome sequences of human immunodeficiency virus type 1 subtypes G and A/G intersubtype recombinants. Virology.

[30] Lengauer T, Sander O, Sierra S, Thielen A, Kaiser R (2007). Bioinformatics prediction of HIV coreceptor usage. Nat Biotechnol.

[31] Billings E, Kijak GH, Sanders-Buell E, Ndembi N, O’Sullivan AM, Adebajo S (2019). New Subtype B Containing HIV-1 Circulating Recombinant of sub-Saharan Africa Origin in Nigerian Men Who Have Sex With Men. J Acquir Immune Defic Syndr (1999).

[32] Murzakova A, Kireev D, Baryshev P, Lopatukhin A, Serova E, Shemshura A, et al. Molecular Epidemiology of HIV-1 Subtype G in the Russian Federation. Viruses. 2019;11(4). 10.3390/v11040348. Epub 2019/04/19. PubMed PMID: 30995717. 10.3390/v11040348 PMC6521041 30995717

[33] Abecasis AB, Wensing AM, Paraskevis D, Vercauteren J, Theys K, Van de Vijver DA (2013). HIV-1 subtype distribution and its demographic determinants in newly diagnosed patients in Europe suggest highly compartmentalized epidemics. Retrovirology..

[34] Hernando V, Alvarez-del Arco D, Alejos B, Monge S, Amato-Gauci AJ, Noori T (2015). HIV infection in migrant populations in the European Union and European Economic area in 2007–2012: an epidemic on the move. J Acquir Immune Defic Syndr.

[35] Mir D, Jung M, Delatorre E, Vidal N, Peeters M, Bello G (2016). Phylodynamics of the major HIV-1 CRF02_AG African lineages and its global dissemination. Infect Genet Evol..

[36] Delatorre E, Velasco-De-Castro CA, Pilotto JH, Couto-Fernandez JC, Bello G, Morgado MG (2015). Short Communication: Reassessing the Origin of the HIV-1 CRF02_AG Lineages Circulating in Brazil. AIDS Res Hum Retroviruses.

[37] Baryshev PB, Bogachev VV, Gashnikova NM (2012). Genetic characterization of an isolate of HIV type 1 AG recombinant form circulating in Siberia. Russia. Arch Virol.

[38] Bonsignori M, Hwang KK, Chen X, Tsao CY, Morris L, Gray E (2011). Analysis of a clonal lineage of HIV-1 envelope V2/V3 conformational epitope-specific broadly neutralizing antibodies and their inferred unmutated common ancestors. J Virol..

[39] Hraber P, Korber BT, Lapedes AS, Bailer RT, Seaman MS, Gao H (2014). Impact of clade, geography, and age of the epidemic on HIV-1 neutralization by antibodies. J Virol..

[40] Moore JP, Cao Y, Leu J, Qin L, Korber B, Ho DD (1996). Inter- and intraclade neutralization of human immunodeficiency virus type 1: genetic clades do not correspond to neutralization serotypes but partially correspond to gp120 antigenic serotypes. J Virol..

[41] Dear N, Esber A, Iroezindu M, Bahemana E, Kibuuka H, Maswai J (2022). Routine HIV clinic visit adherence in the African Cohort Study. AIDS Res Ther.

[42] van Schooten J, van Gils MJ (2018). HIV-1 immunogens and strategies to drive antibody responses towards neutralization breadth. Retrovirology.

[43] de Taeye SW, Moore JP, Sanders RW (2016). HIV-1 envelope trimer design and immunization strategies to induce broadly neutralizing antibodies. Trends Immunol..

[44] Blattner C, Lee JH, Sliepen K, Derking R, Falkowska E, de la Pena AT (2014). Structural delineation of a quaternary, cleavage-dependent epitope at the gp41-gp120 interface on intact HIV-1 Env trimers. Immunity..

[45] Gorman J, Soto C, Yang MM, Davenport TM, Guttman M, Bailer RT (2016). Structures of HIV-1 Env V1V2 with broadly neutralizing antibodies reveal commonalities that enable vaccine design. Nat Struct Mol Biol.

[46] McLellan JS, Pancera M, Carrico C, Gorman J, Julien JP, Khayat R (2011). Structure of HIV-1 gp120 V1/V2 domain with broadly neutralizing antibody PG9. Nature..

[47] Pancera M, McLellan JS, Wu X, Zhu J, Changela A, Schmidt SD (2010). Crystal structure of PG16 and chimeric dissection with somatically related PG9: structure-function analysis of two quaternary-specific antibodies that effectively neutralize HIV-1. J Virol..

[48] Krebs SJ, Kwon YD, Schramm CA, Law WH, Donofrio G, Zhou KH (2019). Longitudinal analysis reveals early development of three MPER-directed neutralizing antibody lineages from an HIV-1-infected individual. Immunity.

[49] Robb ML, Eller LA, Rolland M (2016). Acute HIV-1 Infection in Adults in East Africa and Thailand. N Engl J Med..

[50] Walker LM, Simek MD, Priddy F, Gach JS, Wagner D, Zwick MB (2010). A limited number of antibody specificities mediate broad and potent serum neutralization in selected HIV-1 infected individuals. PLoS Pathog.

[51] McCoy LE, McKnight A. Lessons learned from humoral responses of HIV patients. Current opinion in HIV and AIDS. 2017. 10.1097/coh.0000000000000361. Epub 2017/02/17. PubMed PMID: 28207488. 10.1097/COH.0000000000000361 28422783

[52] Awi NJ, Teow SY (2018). Antibody-mediated therapy against HIV/AIDS: Where are we standing now?. J Pathog.

[53] Xu L, Pegu A, Rao E, Doria-Rose N, Beninga J, McKee K (2017). Trispecific broadly neutralizing HIV antibodies mediate potent SHIV protection in macaques. Science..

[54] Wieczorek L, Sanders-Buell E, Zemil M, Lewitus E, Kavusak E, Heller J (2023). Evolution of HIV-1 envelope towards reduced neutralization sensitivity, as demonstrated by contemporary HIV-1 subtype B from the United States. PLoS Pathog.

[55] Bournazos S, Klein F, Pietzsch J, Seaman MS, Nussenzweig MC, Ravetch JV (2014). Broadly neutralizing anti-HIV-1 antibodies require Fc effector functions for in vivo activity. Cell..

[56] Mascola JR, Louder MK, Surman SR, Vancott TC, Yu XF, Bradac J (1996). Human immunodeficiency virus type 1 neutralizing antibody serotyping using serum pools and an infectivity reduction assay. AIDS Res Hum Retroviruses.

[57] Gray GE, Huang Y, Grunenberg N, Laher F, Roux S, Andersen-Nissen E, et al. Immune correlates of the Thai RV144 HIV vaccine regimen in South Africa. Science translational medicine. 2019;11(510). 10.1126/scitranslmed.aax1880. Epub 2019/09/20. PubMed PMID: 31534016. 10.1126/scitranslmed.aax1880 PMC7199879 31534016

