# Supplementary figures and images for: Differences in neutralizing antibody sensitivities and envelope characteristics indicate distinct antigenic properties of Nigerian HIV-1 subtype G and CRF02_AG
Source: Virol J. 2024 Jun 29;21:148. doi: 10.1186/s12985-024-02394-y (PMC11218331; doi:10.1186/s12985-024-02394-y)

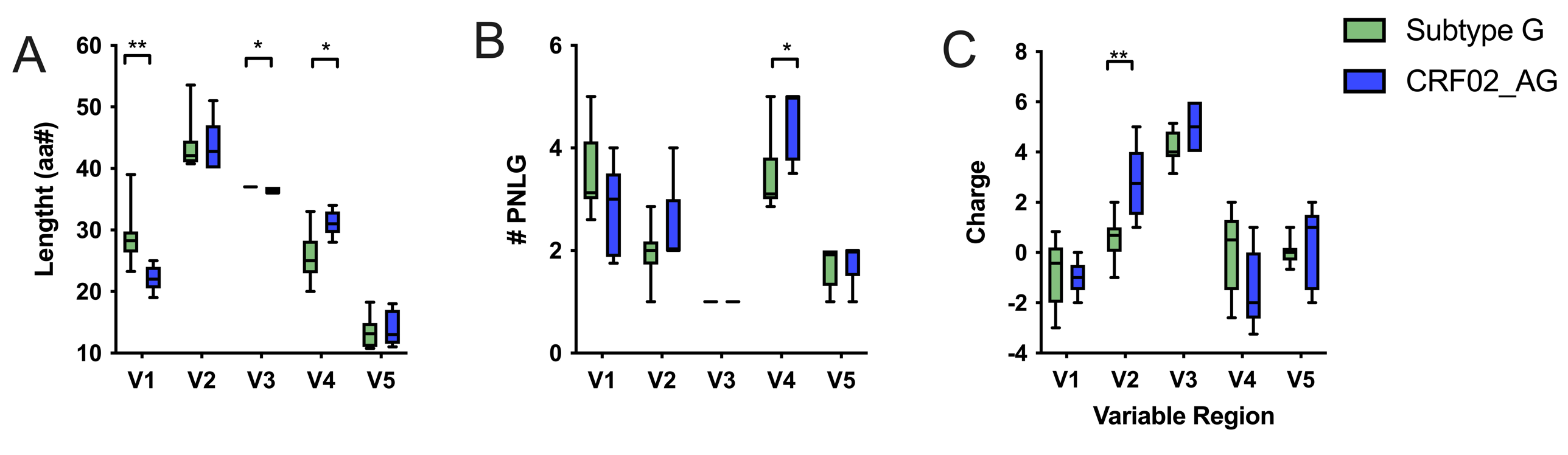

Supplement: Supplementary file 2 — Supplementary Material 2: Fig. S1. Antigenic characteristics of Nigerian subtype G and CRF02_AG Envs shown by averaged values per participant. A geometric mean value for all sequences for each participant was derived and graphed for: A) Variable loop length, B) number of potential N-linked glycosylation sites (PNLG), and C) overall charge, and presented for subtype G (green) and CRF02_AG (blue) Envs. Statistical differences were determined using Mann Whitney U Test; * = p<0.05, ** = p<0.005, *** = p<0.0005. [file 12985_2024_2394_MOESM2_ESM.jpg]

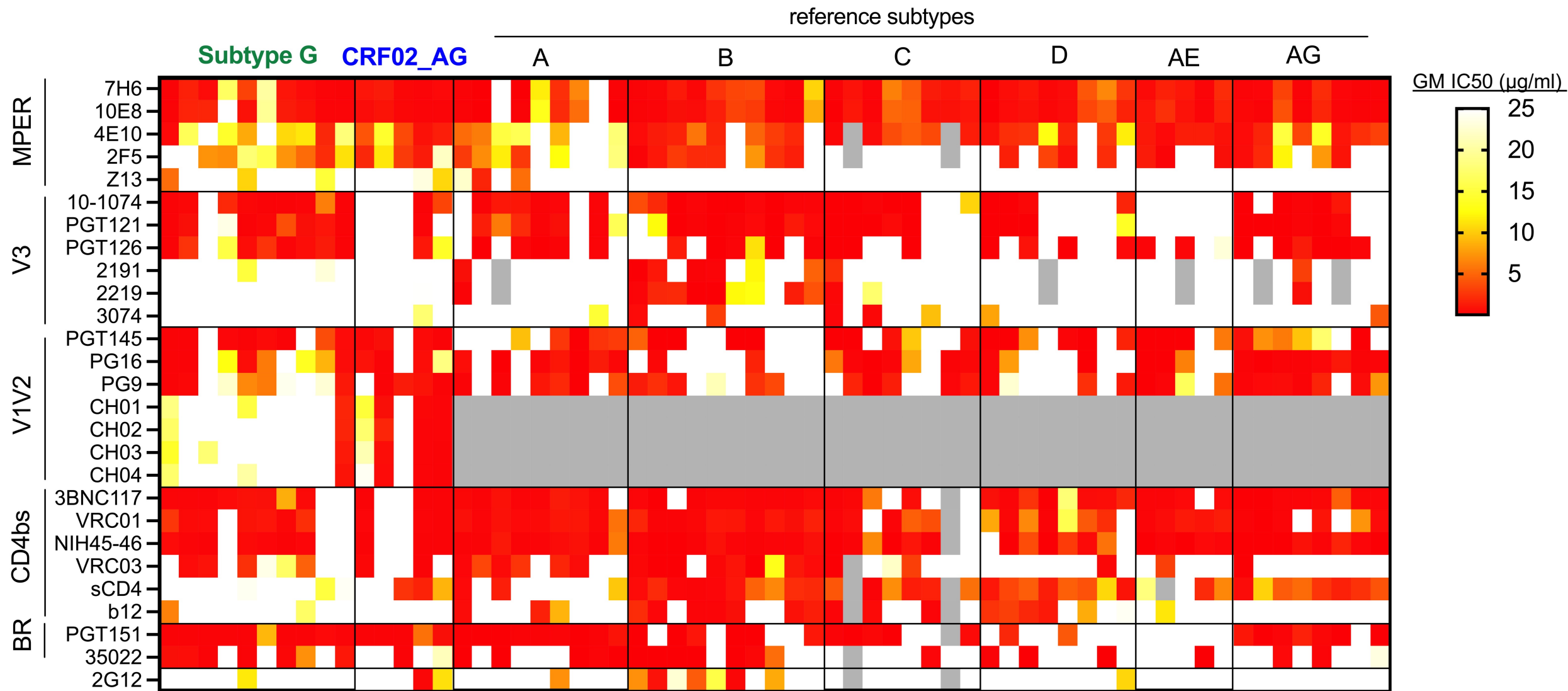

Supplement: Supplementary file 3 — Supplementary Material 3: Fig. S2. Heat map of the NmAb neutralization of subtype G and CRF02_AG GM IC50s compared with reference panel PSV IC50s. The IC50s for each NmAb against all of the clones from each participant were used to generate a GM IC50 and a heat map of these values was generated. The clone subtypes or CRFs are indicated at the top of the figure and the NmAbs and Env domains are listed to the left. As indicated by the scale, stronger red coloring indicates more potent neutralization; grey shading denotes not tested. [file 12985_2024_2394_MOESM3_ESM.pdf]

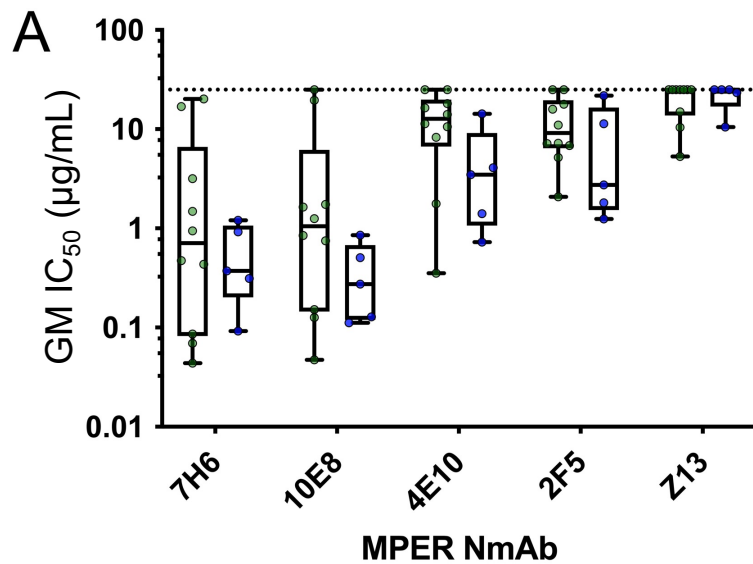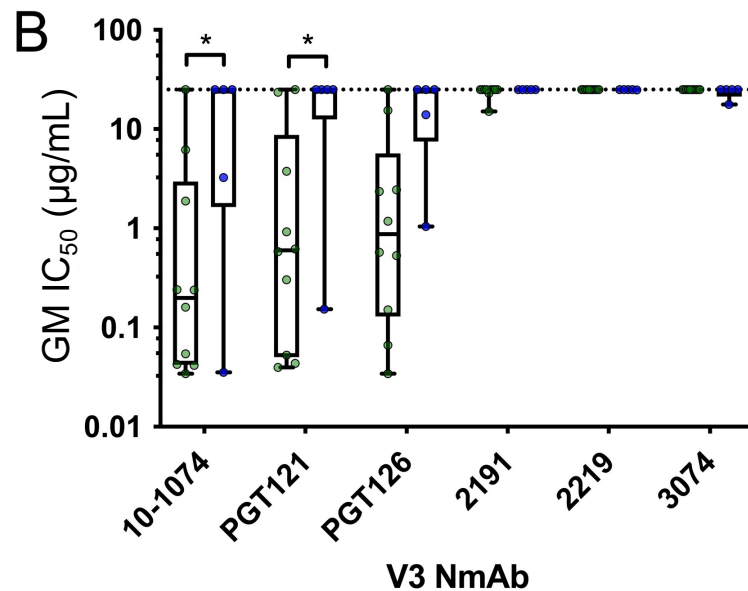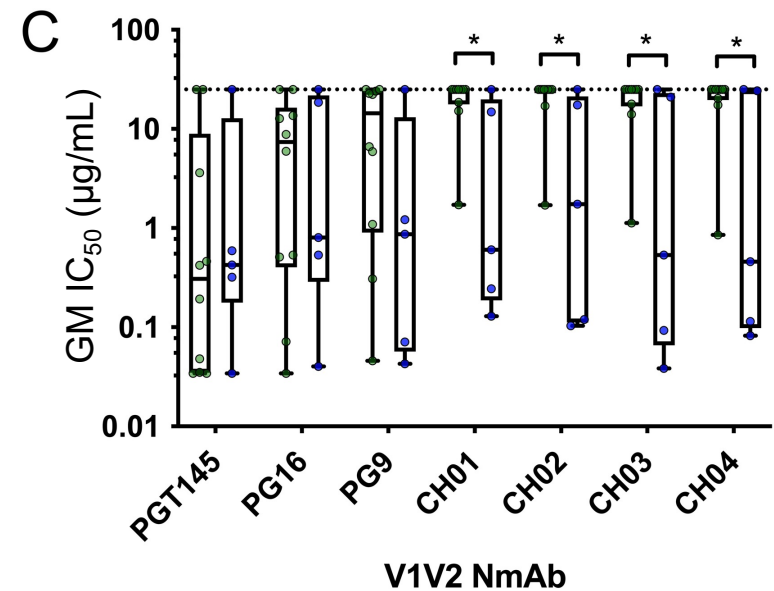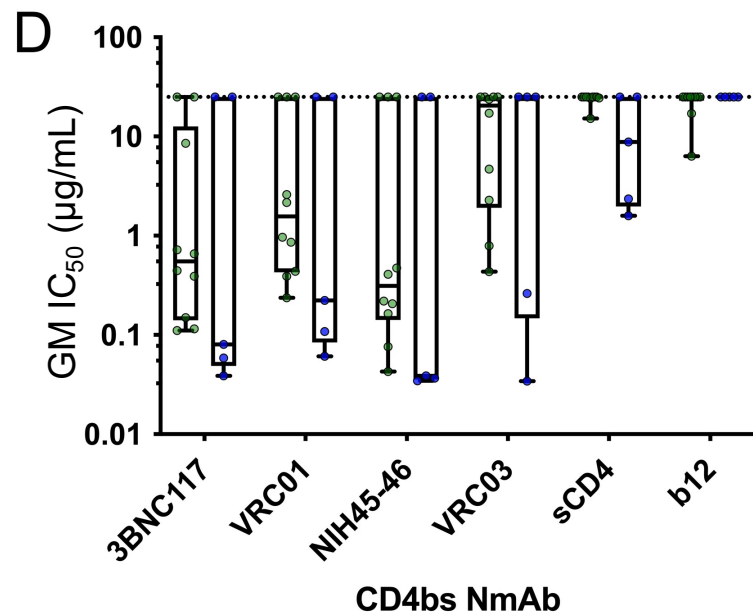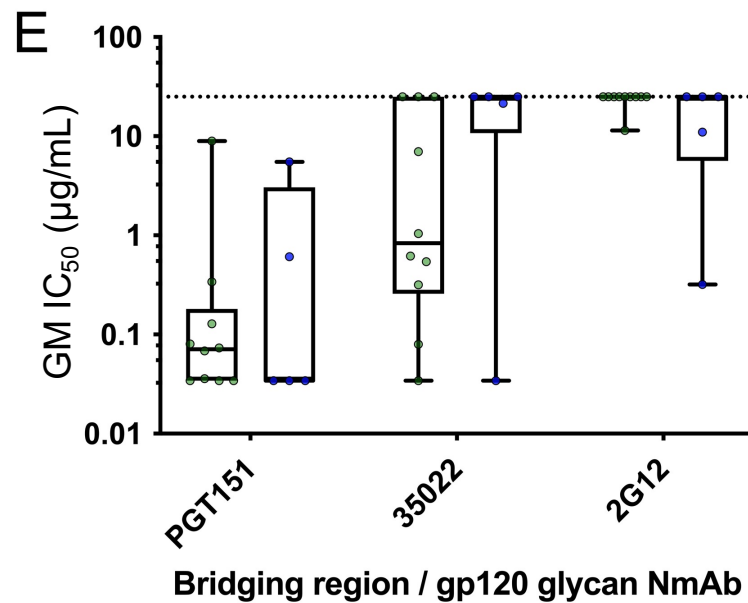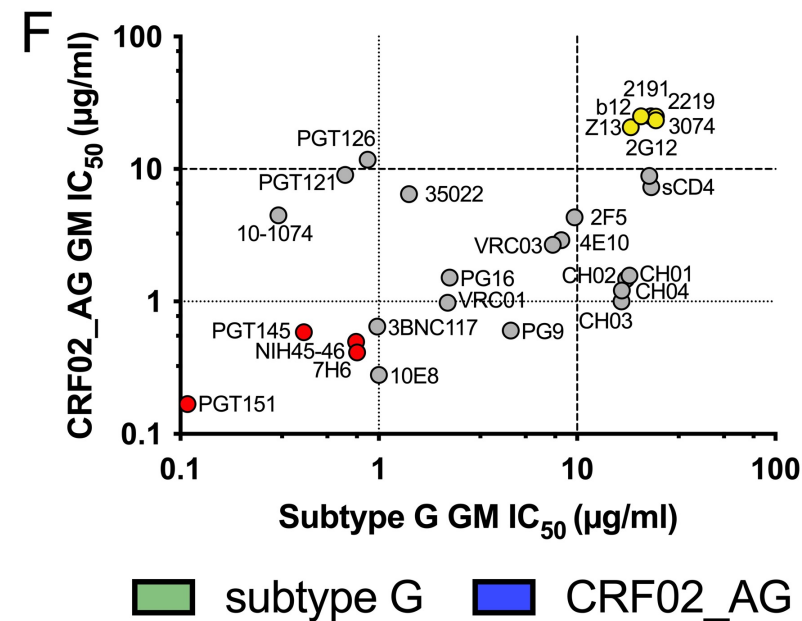

Supplement: Supplementary file 4 — Supplementary Material 4: Fig. S3. Neutralization profiles for subtype G versus CRF02_AG using individual NmAbs and GM IC50s for all clones per participant. NmAbs targeting the A) MPER, B) V3, C) V1V2, D) CD4bs and E) bridging regions and gp120 glycan were tested and GM IC50s were generated for all clones for each participant and graphed. Statistical differences between Subtype G and CRF02_AG PSV were determined using Mann Whitney U Test; *= p<0.05, ** = p<0.005, *** = p<0.0005, as indicated. F) The NmAb GM IC50 was determined for all subtype G or CRF02_AG PSV and plotted to reflect relative NmAb potencies and differences between HIV subtype G and CRF02_AG. The dotted and dashed lines indicate NmAb neutralization potency of GM IC50 = 1 mg/ml or 10 mg/ml, respectively. NmAbs shown in red circles are potently neutralizing against both subtype G and CRF02_AG; NmAbs shown in yellow were weakly neutralizing against both. [file 12985_2024_2394_MOESM4_ESM.pdf]
